# Rationing in healthcare—a scoping review

**DOI:** 10.3389/fpubh.2023.1160691

**Published:** 2023-06-21

**Authors:** Jakub Berezowski, Michał Czapla, Stanisław Manulik, Catherine Ross

**Affiliations:** ^1^National Institute of Geriatrics, Rheumatology and Rehabilitation, Warsaw, Poland; ^2^Institute of Heart Diseases, University Hospital, Wroclaw, Poland; ^3^Department of Emergency Medical Service, Wroclaw Medical University, Wroclaw, Poland; ^4^Group of Research in Care (GRUPAC), Faculty of Health Science, University of La Rioja, Logroño, Spain; ^5^Department of Nursing and Obstetrics, Faculty of Health Sciences, Wroclaw Medical University, Wroclaw, Poland; ^6^The Centre for Cardiovascular Health, School of Health and Social Care, Edinburgh Napier University, Edinburgh, United Kingdom

**Keywords:** priority setting, helathcare, rationing, economy, health economic

## Abstract

Healthcare rationing has been the subject of numerous debates and concerns in the field of health economics in recent years. It is a concept which refers to the allocation of scarce healthcare resources and involves the use of different approaches to the delivery of health services and patient care. Regardless of the approach used, healthcare rationing fundamentally involves withholding potentially beneficial programs and/or treatments from certain people. As the demands placed on health services continue to rise and with that significant increases to the cost, healthcare rationing has become increasingly popular and is deemed necessary for the delivery of affordable, patient-care services. However, public discourse on this issue has largely been centered on ethical considerations with less focus on economic rationality. Establishing the economic rationality of healthcare rationing is essential in healthcare decision-making and consideration of its adoption by healthcare authorities and organizations. This scoping review of seven articles demonstrates that the economic rationality of healthcare rationing is the scarcity of healthcare resources amidst increased demand and costs. Therefore, supply, demand, and benefits are at the core of healthcare rationing practices and influence decisions on its suitability. Given the increased costs of care and resource scarcity, healthcare rationing is a suitable practice towards ensuring healthcare resources are allocated to people in a rational, equitable, and cost-effective manner. The rising costs and demands for care place significant pressure on healthcare authorities to identify suitable strategies for the allocation of healthcare resources. Healthcare rationing as a priority-setting strategy would support healthcare authorities identify mechanisms to allocate scarce resources in a cost-effective manner. When used in the context of a priority-setting approach, healthcare rationing helps healthcare organizations and practitioners to ensure that patient populations achieve maximum benefits at reasonable costs. It represents a fair allocation of healthcare resources to all populations, especially in low-income settings.

## 1. Introduction

Rationing in healthcare is a concept that refers to the allocation of scarce resources, which involves withholding potentially beneficial treatment from certain individuals or groups of people (1). This idea has gained traction in recent years due to the scarcity of healthcare resources amidst a background of increases in demand. While rationing seems essential because of the scarcity of health resources, it has generated controversies. While rationing is unavoidable even in industrialized countries, it also raises controversies due to its potential impacts on the lives of the affected individuals or population groups. This scoping review explores the economic rationality of rationing in healthcare amidst the increasing costs of care.

Healthcare rationing has remained a controversial issue over the past decades despite the fact that it is considered necessary in the modern healthcare sector. Scheunemann and White (1) define healthcare rationing as the allocation of scarce healthcare resources to different populations. Even though this concept is defined in different ways by different groups, there is a general consensus that it involves denying some patients potentially beneficial treatments on the basis of scarcity. The concept of rationing in healthcare is rooted in the idea of priority setting, a process that involves ranking alternative healthcare programs and services based on normative and technical rules (2). Through ranking different programs and treatments, priority setting primarily involves allocating scarce resources. Resource allocation remains a major problem for health systems across the globe as in some areas the demand for services is outstripping the capacity to supply them. Transparency in decision making processes in the context of healthcare rationing has proved controversial but is critical and there remains scope to improve the effectiveness of this transparency (3, 4).

Frenk and Moon (5) described the global health system as unstructured, complex and pluristic, lacking in effective coordination and governance, with a number of actors with different vested interests. They further highlight that examples of major influencers including the World Trade Organisation play a significant role in the monetarization of the system. These factors further increase the complexity of the landscape. Healthcare organizations across the globe are facing numerous challenges relating to resource shortage, increasing costs, and increasing demand. The COVID-19 pandemic increased the need of such rationing beyond what has previously been utilized and placed unmitigated pressure on health services, bringing an increase need to ration resources to almost every countries health system. This has placed the topic in the spotlight of the public and with that raised questions regarding the ethical and transparent approaches employed in its execution. Due to these challenges, organizations are looking for measures to obtain the best value from their programs and services. Priority setting and rationing are strategies considered and adopted by healthcare policymakers and administrators to support organizations obtain the best value for money from scarce healthcare resources. According to the World Health Organization, rationing in healthcare is a prerequisite for universal health coverage (6). Despite being considered necessary for universal health coverage, rationing remains controversial. Stakeholders within and outside the public health sector continue to raise concerns regarding this practice. Debates and discussion regarding this practice need to also examine the economic rationality of rationing in healthcare. In some cases literature shows that rationing of health care denying of patients for economic benefit/limited access with care by any means (6). Amidst the rising healthcare costs and increase in disease burden, the issue of healthcare rationing should be examined from an economic perspective. Economic assessment of healthcare rationing would help to provide a suitable decision-making framework for healthcare organizations and practitioners. The topic of rationing in medicine has been discussed for many years, but there are no papers appearing that accurately document this phenomenon. In the last few years, there have been reports on rationing in nursing care. Not only are definitions and research tools in this area being discussed, but studies on rationing in nursing care are being conducted (7–9). The reported findings indicate that rationing influences patient safety and care outcomes. It depends not only on the resources available in the hospital, but also on the personality of the nurses (10–12).

The aim of this scoping review was to explore the economic rationality of rationing in healthcare amidst the increasing costs of care. The review examines existing peer-reviewed literature with a view of identifying knowledge gaps which may support the decision-making strategies of healthcare leaders and practitioners. The following research questions are investigated in this scoping review:

1. What is the economic rationality of rationing in healthcare?
2. Is it adequate enough support the adoption of this practice by healthcare organizations?

## 2. Methods

To answer the research questions, a systematic review of the economic perspective of rationing in healthcare was conducted. A scoping review was adopted for this study since it entails a broader search strategy that allows transparency, reliability, and reproducibility of literature relating to the topic.

### 2.1. Search strategy

Since a scoping review design is employed for this study, the identification of relevant studies involves the use of a broad search strategy. The relevant studies for this scoping review are original peer-reviewed journals published in the English language over the past 7 years. The search for relevant literature was conducted in three electronic databases: EBSCOhost, CINAHL, and PubMed. Some of the keywords and phrases used in the search include: “rationing in healthcare,” “economic rationality of healthcare rationing,” “rationing by healthcare organizations,” “financial perspective in healthcare rationing,” “economic challenges for healthcare rationing.”

### 2.2. Inclusion criteria

To be included in the review, the peer-reviewed articles had to meet three criteria. First, the articles must have considered the economic rationality or financial perspective of rationing in healthcare. This implies that studies that did not examine the issue from a financial perspective were excluded from the review. Secondly, only peer-reviewed full articles written in English and published within the last 7 years were considered. As a result, abstracts and studies published in other languages were excluded. Third, grey literature, systematic reviews, narratives, and commentaries were considered this scoping review.

### 2.3. Study selection and data extraction

Following the search on the three electronic databases, search results were screened based on inclusion criteria. Abstracts, duplicates, and articles published in a language other than English were removed. After the identification of relevant studies, data was recorded in a spreadsheet by the reviewer. A narrative account of the data extracted from the studies were prepared to provide insights into the economic rationality of rationing in healthcare.

## 3. Results

**Figure 1:**
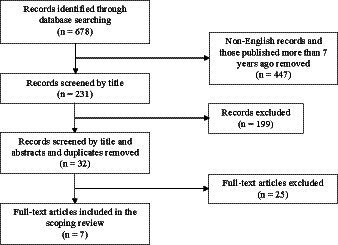
Study identification and selection.

The searches from the three electronic databases generated a total of 678 records, after removal of non-English language papers and those which exceeded the 7 years exclusion period the 231 titles remaining were screened on the basis of title, abstract and duplication as shown in Figure 1. As a result, a total of 32 full-text articles remained from the different libraries. After a further full-text assessment based on inclusion criteria, a total of seven articles were identified and included in the final data extraction. The final articles included in the scoping review for data extraction provided recent findings regarding the economic rationality of rationing in healthcare amidst the ever-increasing costs and demand for care.

### 3.1. Economic issues facing healthcare

Health care rationing has been a source of contentious debate in many countries (13–18). According to Almesned et al. (19), healthcare organizations are faced with significant and challenging economic issues including increasing demands, increased costs, and limited budgets. As a result, economic evaluation is an important component in healthcare decision-making because it helps to identify, compare and value the costs and results of different healthcare policies and programs. These researchers found that economic evaluation is an important part of healthcare financing decision-making. Keliddar et al. (6) contend that limited resources are the major economic issues facing healthcare organizations in today’s society. In concurrence with Almesned et al. (6) and Keliddar et al. (19) note that healthcare resources and financing are increasingly limited at a time when demands are unlimited. The increased shortages in healthcare resources have put more pressure on healthcare practitioners and organizations to generate the best value for money.

Zweifel (20) contends that health economists face challenges relating to how to balance the benefits and costs of healthcare services and programs. On one hand, there is a need to safeguard the costs of healthcare services and programs while ensuring citizens or patients obtain maximum benefits, while on the other, there is a pursuit of profit by some healthcare providers and practitioners. Keliddar et al. (6) state that healthcare authorities are increasingly looking for measures and strategies that would help achieve a balance between costs and benefits. The achievement of such a balance is critical to providing affordable, patient-centered care services with the best outcomes. Similarly, Moosa and Luyckx (21) state that the scarcity of healthcare resources remains a distressing challenge for healthcare authorities. This issue is worse in low-income settings or during periods of economic recession and public spending cuts (6, 21). Public health issues such as the COVID-19 pandemic place significant pressure on the availability of scarce health resources. Some authors see opportunity in this situation for Universal Health Coverage (22).

### 3.2. Economic rationality for healthcare rationing

Almesned et al. (19) opine that the scarcity of health resources and the need for economic evaluation provides the economic foundation for healthcare rationing. They contend that these factors imply that healthcare rationing is unavoidable and would play a critical role in healthcare delivery processes. In this regard, Almesned et al. (19) state that countries across the globe, especially low-income nations, should ration healthcare explicitly. Explicit rationing is an approach in which healthcare resources are distributed by a specific policy (23–25). However, decision-making on the approach of healthcare rationing should be based on the outcomes of economic evaluation (26, 27). According to Hauge et al. (28), explicit priority setting is a suitable approach to healthcare rationing since it ensures transparency and legitimacy regarding rationing decisions. This is also confirmed by the authors of other studies (29–31). Despite raising some concerns, this approach to healthcare rationing provides a rational process for the allocation of scarce healthcare resources. Through this process, healthcare authorities find a suitable balance between the rising costs/demands of care and realizing maximum benefits. However, opine that rationing by waiting is a better approach as it enables profit-maximizing hospitals to create a balance between quality expected outcomes and cost (32, 33). Using this approach, such hospitals attract patients by raising quality while decreasing waiting times.

Zweifel et al. (34) contend that rationing healthcare is necessary for the delivery of affordable, patient-centered care. However, healthcare rationing is justified in situations where major populations do not have access to essential medical care because of an increase in costs. In addition, healthcare rationing is justified in situations where the incomes and profits of healthcare providers increase at a rate that threatens the social fabric of a nation. Keliddar et al. (6) found that the economic rationality of healthcare is supported by three factors, i.e., scarcity, controllability, and value. In this case, healthcare is a scarce commodity and valuable resource whose supply should be rational, cost-effective, and equitable. Healthcare rationing provides a framework for ensuring that the scarce healthcare resources are equitable, rational, and cost-effective (6, 35). These factors imply that healthcare rationing can be adopted by different stakeholders including policymakers, patients, providers, and managers. Therefore, the economic rationality of healthcare rationing is adequate to support its adoption in today’s health sector since it provides a framework for ensuring the fair allocation of scarce resources.

### 3.3. Study limitation

This review was based on an extensive search for studies, and perhaps some related studies were omitted. Furthermore, the quality of the studies included in the review was not assessed and the studies were selected only in terms of access to evidence and answering the question of this study, rather than in terms of strengths and weaknesses. The number of studies on the topic of rationing in healthcare is small.

## 4. Conclusion

In conclusion, healthcare rationing refers to the allocation of scarce healthcare resources to patient populations. This issue has attracted considerable attention in recent years, especially with the increase in healthcare costs at a time when demand is also skyrocketing. However, healthcare rationing has generated concerns among different stakeholders regarding its necessity and impact on healthcare delivery. While ethical principles have dominated public discourse on this matter, the economic rationality of this practice is seemingly ignored. This scoping review establishes that the economic rationality of healthcare rationing is balancing between the rising costs/demands of care and the maximum benefits of scarce healthcare resources. Supply and demand play an important role in healthcare rationing decision-making in consideration of the scarcity of resources. The rising costs and demands for care place significant pressure on healthcare authorities to identify suitable strategies for the allocation of healthcare resources. The economic rationality of healthcare rationing is adequate to support the adoption of this practice by healthcare organizations as it helps to ensure that healthcare resources are distributed in a rational, cost-effective, and equitable manner.

## Author contributions

JB: conceptualization. JB and MC: methodology. JB: software and visualization. JB and SM: formal analysis. JB, SM, and CR: investigation. JB and SM: writing—original draft preparation. JB, MC, and CR: writing—review and editing. CR: supervision. MC: project administration. All authors contributed to the article and approved the submitted version.

## Funding

This research was funded by Ministry of Science and Higher Education of Poland under the statutory grant of the Wroclaw Medical University (SUBZ.E250.23.020).

## Conflict of interest

The authors declare that the research was conducted in the absence of any commercial or financial relationships that could be construed as a potential conflict of interest.

## Publisher’s note

All claims expressed in this article are solely those of the authors and do not necessarily represent those of their affiliated organizations, or those of the publisher, the editors and the reviewers. Any product that may be evaluated in this article, or claim that may be made by its manufacturer, is not guaranteed or endorsed by the publisher.
